# Bioelectrical impedance analysis instruments: how do they differ, what do we need for clinical assessment?

**DOI:** 10.1097/MCO.0000000000001142

**Published:** 2025-07-15

**Authors:** Yves M. Dupertuis, Wedali Jimaja, Cheryle Beardsley Levoy, Laurence Genton

**Affiliations:** a Clinical Nutrition, Geneva University Hospitals; b Faculty of Medicine, University of Geneva, Geneva, Switzerland; c School of Nursing, Tennessee Technological University, Tennessee, USA

**Keywords:** bioelectrical impedance analysis, body composition, electrode configuration, nutritional assessment

## Abstract

**Purpose of review:**

Bioelectrical impedance analysis (BIA) is a widely used, noninvasive method for assessing body composition. Recent technological advances have diversified BIA devices in terms of measurement frequency, electrode configuration, and portability. This review outlines key criteria for selecting a BIA system according to clinical or research needs.

**Recent findings:**

Single-frequency BIA (SF-BIA) devices, typically consumer-grade with hand-to-hand or foot-to-foot configurations, are affordable and easy to use but often lack raw data access, clinical validation, and regulatory certification. In contrast, multifrequency BIA (MF-BIA) systems, especially octopolar models, enable segmental analysis and provide greater accuracy for evaluating fluid distribution and lean mass. However, they are costlier, depend on proprietary algorithms, and generally require standing measurements. In hospital settings, portable MF-BIA devices that allow supine, tetrapolar or octopolar assessments are preferable, particularly for use with bedridden patients. Across all contexts, standardized measurement protocols and access to raw parameters (*Z*, *R*, Xc, PhA) are essential to apply accurate, population-specific predictive equations.

**Summary:**

Reliable use of BIA requires careful consideration of device type, data accessibility, and methodological consistency. Portable, regulatory-certified MF-BIA systems with tetrapolar or octopolar configurations and access to raw data offer the most accurate and adaptable solutions for clinical and research applications.

## INTRODUCTION

Bioelectrical impedance analysis (BIA) is a widely used method for estimating body composition and has emerged as a valuable tool for managing hypercatabolism and fluid balance in various clinical settings. It relies on the measurement of the body's electrical impedance in response to a painless, low-intensity alternating current. BIA is simple, rapid, noninvasive, and provides a practical alternative to more costly and less accessible techniques such as computed tomography (CT), MRI, or dual-energy X-ray absorptiometry (DXA) [1]. It is recommended by the Global Leadership Initiative on Malnutrition (GLIM) for evaluating muscle mass loss [2]. Over time, BIA has gained widespread adoption, with a growing number of devices developed for both clinical and consumer uses, varying significantly in complexity, precision, and cost. As a result, choosing the most suitable BIA device for a given application has become more challenging. This review aims to provide key criteria to guide the selection of BIA technologies and protocols, based on the specific needs of research or clinical practice. 

**Box 1:**
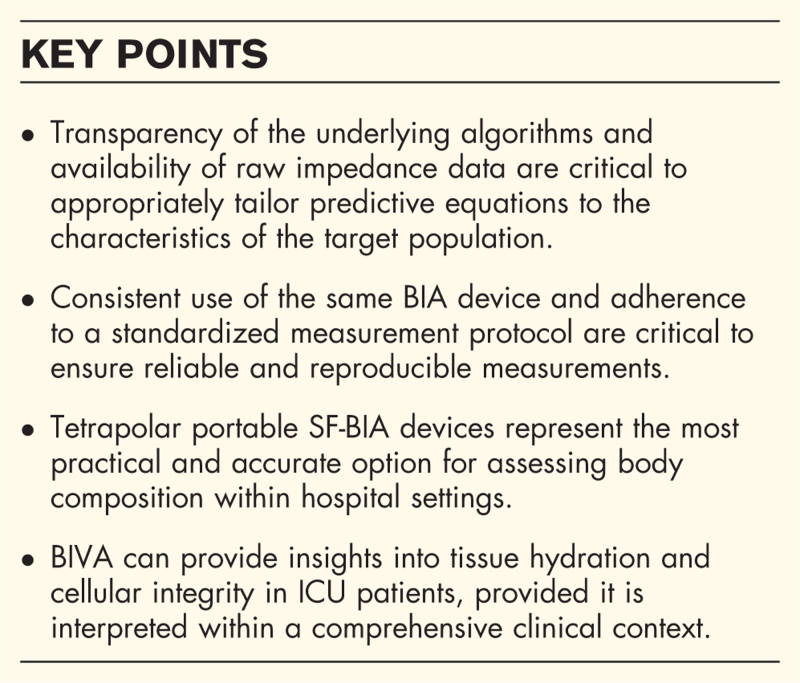
no caption available

## PRINCIPLES OF BIOELECTRICAL IMPEDANCE ANALYSIS MEASUREMENT

BIA estimates body composition by measuring impedance (*Z*) and phase angle (PhA), generated by a low-intensity (<1 mA) alternating current across the body at frequencies ranging from 1 to 1000 kHz. The current flows through conductive tissues and electrolyte-rich fluids and is impeded by adipose tissues. In a normally hydrated individual, impedance increases together with adiposity. Single-frequency BIA (SF-BIA) at 50 kHz allows estimation of fat mass, fat-free mass (FFM), skeletal muscle mass (SMM), and total body water (TBW), using predictive equations incorporating sex, age, height, and weight. Multifrequency BIA (MF-BIA) improves accuracy by differentiating intracellular water (ICW) from extracellular water (ECW), exploiting the frequency-dependent permeability of cell membranes. PhA, reflecting cell membrane capacitance, is a marker of membrane integrity and a prognostic indicator in chronic diseases like cancer, HIV, and renal failure. Some devices report resistance (*R*) and reactance (Xc), from which *Z* and PhA are derived.


$$Z\;=\;\sqrt{R^{2}+Xc^{2}}\;PhA(\;{}^{\circ})\;=\;\arctan\;\left(\frac{Xc}{R}\right)x\left(\frac{180}{\pi }\right)$$

## WHICH PREDICTIVE EQUATION TO CHOOSE?

Numerous predictive equations have been developed through cross-validation with reference methods such as CT, MRI, DXA, total-body potassium (TBK), air displacement plethysmography (ADP), the four-compartment model (4C), and isotopic dilution techniques using deuterium oxide (D_2_O) or sodium bromide (NaBr). However, these so-called criterion methods are themselves indirect assessments of body composition, each with varying degrees of precision depending on the compartment measured. For instance, DXA, ADP, and 4C models are considered accurate for estimating fat mass, FFM, and lean soft tissue mass; D_2_O and NaBr dilution are used for quantifying TBW and ECW, respectively; TBK for body cell mass; and MRI or CT for SMM. The validity of BIA-derived predictive equations, therefore, strongly depends on the specific criterion method employed, as well as the sample size and demographic characteristics of the population used in their development. For example, Rojano-Ortega *et al*. [3] evaluated the accuracy of manufacturers’ equations of two BIA devices and concluded that they were not a good option for fat mass percent (FM%) assessment compared to new regression equations developed using DXA. Consequently, the results provided by BIA devices that rely on proprietary, undisclosed equations may not be suitable for a specific patient population. Therefore, access to raw data (*Z*, PhA, *R*, Xc) is essential to allow researchers and clinicians to apply the most appropriate and validated predictive equations for their target population. Campa *et al.*[4^▪▪^] classified 106 predictive equations according to the subject's characteristics, so that practitioners now have an updated list of predictive equations for assessing body composition using BIA.

## TYPES OF BIOELECTRICAL IMPEDANCE ANALYSIS INSTRUMENTS AND KEY DIFFERENCES

**FIGURE 1:**
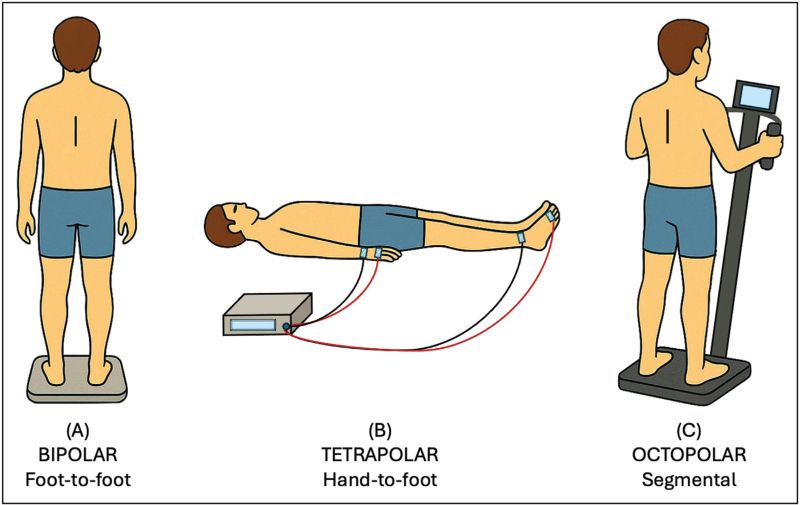
Bioelectrical impedance analysis devices vary in measurement configurations: (a) consumer-grade models typically use a bipolar foot-to-foot or hand-to-hand configuration with a single pair of current-injecting (signal) and voltage-sensing (detection) electrodes; (b) clinical-grade devices use a tetrapolar hand-to-foot configuration with two pairs of signal and detection electrodes, enhancing accuracy by reducing contact resistance between electrodes; (c) advanced systems feature an octopolar arrangement, with a pair of signal and detection electrodes on each hand and foot, allowing segmental assessment of fluid and lean mass distribution.

Commercially available BIA devices differ across several technical and operational features. Measurement techniques include SF-BIA, MF-BIA, bioelectrical impedance vector analysis (BIVA), and bioelectrical impedance spectroscopy (BIS). Electrode configurations may be bipolar, tetrapolar, or octopolar (Fig. 1). Additionally, devices vary considerably in terms of size, portability, connectivity, and cost. In recent years, several studies have assessed the performance of various BIA devices across different clinical settings, highlighting their respective advantages and limitations.

### Single-frequency bioelectrical impedance analysis

SF-BIA devices typically operate at a fixed frequency of 50 kHz to estimate body composition. They are widely used because of their ease of use, portability, and affordability. These devices provide rapid assessments, making them particularly well suited for outpatient consultations. Their use is common in rehabilitation centres and nutrition clinics. Numerous predictive equations validated in specific patient populations are based on 50 kHz SF-BIA measurements to calculate fat mass, FFM, and SMM, which supports their relevance for assessing malnutrition in hospital settings. Recently, a methodology integrating SF-BIA with ultrasound imaging has been developed to quantify total visceral adipose tissue, obviating the need for MRI, DXA, or CT [5]. SF-BIA measurements are, however, sensitive to factors such as hydration status, body temperature, and patient positioning. Overall, SF-BIA provides a rapid and reasonably accurate estimation of fat mass but is supposed to be less effective than MF-BIA in assessing hydrated tissues such as FFM and SMM, particularly in patients with significant alterations in body composition, such as those with severe obesity or cachexia.

### Bioelectrical impedance vector analysis (BIVA)

To avoid the use of unsuitable predictive equation for a specific population, it has been proposed to perform a vector analysis of the raw data (*Z*, PhA, *R*, Xc), standardized with respect to the individual's height. BIVA does not predict water compartments but analyses water and cellular quality from the vector position on a bivariate graph. The position and length of these vectors provide qualitative insights into the individual's hydration and cellular health. A shorter vector indicates fluid overload, while a longer vector suggests dehydration. Recent studies have highlighted BIVA's utility in various populations, including athletes and patients with chronic conditions, emphasizing its role in monitoring physiological changes without relying on population-specific equations [6]. This approach is of particular interest to monitor hypercatabolism and hydration in oncology, ICU, and geriatrics. However, it requires specialized expertise to interpret impedance vectors limiting its use in routine clinical practice. Recent findings confirm that BIVA is a valid method to assess SMM and ICW in active young men and athletes compared to MF-BIA and BIS [7]. However, others report only moderate agreement between raw bioelectrical values and urine-specific gravity [8], indicating that the use of BIVA to assess hydration status in patients still requires further validation.

### Multifrequency bioelectrical impedance analysis

MF-BIA devices use multiple frequencies to enhance the differentiation between body water compartments. They provide estimates for body fluid changes, which are particularly valuable in clinical settings such as nephrology and malnutrition. A recent study showed that detection of lower limb muscle mass changes using MF-BIA is a valuable tool for early diagnosis of sarcopenia in elderly adults [9^▪^]. However, these more expensive BIA devices require standardized conditions for reliable measurements and rely largely on proprietary, undisclosed equations.

### Bioelectrical impedance spectroscopy

BIS assesses body composition by measuring the body's impedance across a spectrum of frequencies, enabling ICW and ECW differentiation. This method provides accurate assessment of hydration status when compared with isotopic dilution method [7] or cumulative fluid balance (CFB) [10], potentially supporting its use in clinical and research settings. Recent studies have also demonstrated the efficacy of BIS in various applications, such as dialysis [11^▪▪^], and the monitoring of adipose tissue changes using new anthropometric multivariate regression models [12].

### Bipolar bioelectrical impedance analysis

BIA devices can employ various electrode configurations to estimate body composition (Fig. 1). Consumer-grade models typically use a single pair of signal and detection electrodes in hand-to-hand or foot-to-foot configurations. The former requires holding a portable monitor with both hands placed on electrodes, while the latter involves standing on a scale with plantar electrodes. Some devices also support foot-to-hand measurements via retractable hand-held electrodes [13]. Compared to octopolar BIA devices, a foot-to-foot device has recently shown significant practicability and potential in assessing lower limb muscle mass as sarcopenia indicator [9^▪^]. These affordable and user-friendly devices have gained popularity, but their reliance on proprietary algorithms limits their transparency and clinical utility. Moreover, they tend to overlook or underestimate excess fat mass in the abdominal region. Siedler *et al.*[14] reported wide variability in the cross-sectional and longitudinal validity and reliability of such devices. Consequently, the utility of foot-to-foot BIA devices for monitoring body composition over time depends heavily on the specific model, individual's body shape, and standardized measurement conditions [9^▪^,^14^]. For greater accuracy in clinical and research settings, tetrapolar or octopolar configurations are preferred.

### Tetrapolar bioelectrical impedance analysis

**FIGURE 2:**
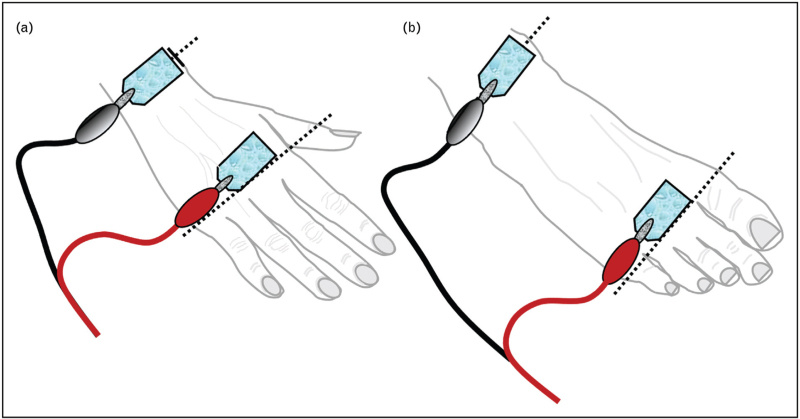
Schematic representation of adhesive electrode placement for tetrapolar and ipsilateral bioelectrical impedance analysis in the supine position: (a) upper limb: the current-injecting (signal) electrode is placed over the metacarpophalangeal joints of the second and third digits, while the voltage-sensing (detection) electrode is positioned proximally over the styloid processes of the radius and ulna; (b) lower limb: the signal electrode is applied over the metatarsophalangeal joints of the second and third toes, and the detection electrode is located midway between the medial and lateral malleoli (ankle bones), over the tibiotalar region.

The classical approach uses an ipsilateral tetrapolar configuration, with two pairs of single-use signal and detection electrodes placed on the dominant-side hand and foot [15]. Unlike hand-to-hand or foot-to-foot configurations, this setup is suitable for bedridden patients and enables whole-body impedance measurement with minimal influence from variations in adipose tissue distribution [16]. Accurate and reproducible data require a standardized protocol, including a minimum 5 cm spacing between electrodes (Fig. 2). Electrode type also affects measurement accuracy; those with a gel contact area at least 400 mm^2^ are recommended, particularly when raw PhA values are used to assess malnutrition risk [17]. Although manufacturers provide validated electrodes, alternatives, such as ECG electrodes, may be cost-effective in clinical settings. However, consistency in electrode type is essential for data reproducibility in clinical monitoring and multicentre studies. A new handheld BIA device enabling tetrapolar measurement without electrodes has also been developed, but its use may present some challenges in older inpatients [18].

### Octopolar bioelectrical impedance analysis

Advanced MF-BIA devices enable segmental analysis via an octopolar configuration, with signal and detection electrodes placed on each hand and foot, allowing impedance measurement of limbs and trunk across multiple frequencies [19]. These systems offer assessment of FFM and fluid distribution, making them valuable in nephrology, sports medicine, and in detecting clinical asymmetries such as muscle atrophy [20] or lymphedema [21]. Octopolar MF-BIA systems have demonstrated high reliability for longitudinal monitoring [14,19]. However, they tend to underestimate FM% and overestimate FFM%, with proportional bias in ECW/TBW ratio compared to reference methods [12,22,23]. Additionally, these devices are often costly and stationary, and do not always provide access to raw impedance data. Furthermore, they require a standing position for measurement, limiting their use in bedridden patients, unlike tetrapolar systems.

### Wearable and connected bioelectrical impedance analyzers

Technological advances have enabled the development of wearable, connected BIA devices, including smartphone-integrated systems and smart wristbands. These tools facilitate real-time body composition monitoring and enhance at-home follow-up. However, significant accuracy discrepancies remain when compared to criterion methods, and further validation is necessary [24].

## CLINICAL ADVANTAGES AND LIMITATIONS

In recent years, BIA has expanded its clinical scope for assessing adiposity, muscle wasting, and hydration across diverse patient groups.

### Fat mass and adipose tissue distribution

In obesity, endocrinology, and weight-management, BIA provides a cost-effective and fast alternative to DXA for total and regional fat mass estimation [5]. SF-BIA enables frequent monitoring but underestimates visceral adiposity and loses accuracy at extreme body sizes. MF-BIA and BIS improve trunk-vs.-limb differentiation, though their precision depends on individual's high-waist circumference and posture (i.e. supine, standing, and seated) [25]. BIVA sensitively detects changes in cell-membrane integrity during weight loss but does not quantify fat mass directly. In outpatients, bipolar electrode configurations facilitate rapid assessment. However, tetrapolar configuration should be preferred to enhance reproducibility during routine follow-up. Octopolar configuration may deliver better segmental adipose profiles but is often impracticable for bedridden patients.

### Muscle wasting monitoring

BIA is gaining recognition as a valuable tool for monitoring hypercatabolism in oncology [26], ICU [27], and geriatrics [28], given the strong association between low PhA values and adverse clinical outcomes, including reduced survival. In these settings, tetrapolar SF-BIA enables rapid bedside screening of SMM, although fluid disturbances, such as ascites or oedema, may lead to overestimation. MF-BIA and BIS partition ICW/ECW for more accurate SMM assessment but require complex modelling. BIVA enables equation-free tracking of cellular depletion without volume outputs [29,30]. In these settings, tetrapolar electrode configuration is preferred for reproducibility, while octopolar configuration enables segmental muscle mapping but is dependent on proprietary undisclosed equations and often impractical for bedridden patients. A recent comprehensive review summarizes the latest technological developments in BIA, highlighting its strengths and limitations in assessing obesity and sarcopenia [31^▪▪^].

### Hydration status and fluid management

In ICU [10], nephrology [11^▪▪^], cardiology [32], neonatology [33], and sport medicine [34], SF-BIA can detect TBW shifts, while MF-BIA and BIS distinguish ECW from ICW, although acquisition times of up to 20 min may interfere with urgent decision-making. BIVA provides real-time qualitative monitoring of fluid status via vector migration [10]. Across applications, tetrapolar configurations balance speed and accuracy [35], whereas octopolar devices afford detailed segmental estimates mapping but are less practical for bedridden patients. A recent study evaluating a wearable BIA sensor in haemodialysis patients demonstrated a strong correlation with ultrafiltration volume (*r* = 0.82), highlighting its potential for monitoring fluid shifts during treatment [36]. However, current evidence does not support the exclusive use of BIA to guide intravenous hydration or volume removal decisions [37]. A recent narrative review highlights the potential of BIA in assessing dynamic fluid shifts and tissue integrity in ICU patients [38^▪▪^].

## CLINICAL REQUIREMENTS

### Regulatory and technical requirements

Clinical assessment of body composition using BIA requires strict adherence to methodological and regulatory standards to ensure reliable results. BIA devices are subject to varying regulations worldwide. In the European Union, they must bear CE marking as Class IIa medical devices; in the United States, they require Food and Drug Administration (FDA) Class II clearance through the 510(k) process. Other regions, such as Canada, Brazil, China, Japan, and India, require device registration, local representation, and compliance with applicable quality standards. Verifying regulatory compliance is essential before acquiring a BIA device for clinical use.

### Accuracy and validation

The clinical accuracy of BIA devices must be demonstrated through validation against criterion methods for both body fat and water compartments [23]. In research settings, access to raw impedance parameters (*R* and Xc or *Z* and PhA), preferably at 50 kHz, is essential for the development and use of population-specific predictive equations [3]. These equations critically influence the estimation of body composition parameters [12].

### Transparency and consistency in device use

Transparency in the algorithms used by BIA devices is crucial for scientific applications. As raw data and outputs can vary significantly between devices, the same BIA system should be used throughout both cross-sectional and longitudinal assessments to ensure data comparability [39]. For hospital-based research and clinical applications, a tetrapolar or octopolar MF-BIA device allowing supine measurement and access to raw data is preferred, particularly for use in bedridden patients. Table 1 lists examples of medical-grade devices with tetrapolar or octopolar configurations recently used in clinical studies.

**Table 1:** Nonexhaustive list of clinical-grade bioelectrical impedance analysis devices according to tetrapolar, or octopolar measurement mode, frequencies, possibility of carrying out measurement in supine position, raw data availability, validation with criterion methods, and indicative price

BIA device	Mode	Frequency (kHz)	Supine	Raw data	Validation	Price (€ VAT included)	Ref.
Akern BIA 101	Tetra–Octo	50	Yes	*R*, Xc, PhA	DXA, CT, MRI, D_2_O, NaBr, 4C	4000–7000	[3,29,33]
Biody XPERT^ZM^	Tetra	1–1000	Yes	*R*, Xc, *Z*, PhA	DXA	5000	[18]
BodyStat MultiScan 5000	Tetra	3–1000 (50 freq.)	Yes	*R*, Xc, *Z*, PhA	D_2_O, DXA, CFB	6000–10 000	[10]
DataInput Nutriguard	Tetra	5, 50, 100	Yes	*R*, Xc, *Z*, PhA	DXA, 40K	3000	[13,17]
2Human Im Touch	Tetra	5, 50, 100, 250	Yes	*R*, Xc, *Z*, PhA	DXA	3000	[6]
ImpediMed SFB7	Tetra	3–1000 (256 freq.)	Yes	*R*, Xc, *Z*, PhA	DXA, ADP, TBK, D_2_O	6000	[7]
InBody 270	Octo	20, 100	No	PhA	DXA	3000–6000	[9^▪^]
InBody 770	Octo	1, 5, 50, 250, 500, 1000	No	*Z*, PhA	DXA, 4C	20 000	[8,9^▪^,^19^,^23^]
InBody S10	Octo	1, 5, 50, 250, 500, 1000	Yes	*Z*, PhA	DXA	15 000	[25]
Maltron BioScan	Tetra	5, 50, 100, 200	Yes	*R*, Xc, *Z*, PhA	DXA, ADP	14 000	[16]
Seca mBCA 515	Octo	1–1000 (19 freq.)	No	*R*, Xc, *Z*, PhA	DXA, ADP, MRI, D_2_O, 4C	12 000	[14,22]
Tanita BC418	Octo	50	No	*R*, Xc, *Z*, PhA	DXA, 4C	4000–6000	[9^▪^]
Tanita MC-780-MA	Octo	5, 50, 250	No	/	DXA	7000	[9^▪^,^12^]

BIA, bioelectrical impedance analysis; PhA, phase angle; *R*, resistance; Xc, reactance; *Z*, impedance.

### Standardization of measurement protocols

To minimize inter-individual and intra-individual variability, BIA protocols must be rigorously standardized. Measurements can be greatly affected by the subject's posture (standing, sitting, and supine) [40]. Therefore, consistency in positioning and device use is critical during longitudinal follow-up. Physiological factors such as hydration status, skin temperature, recent physical activity, and the use of topical products can also affect tissue conductivity and distort results [17]. Table 2 summarizes the key conditions required to ensure reproducibility and comparability of raw data.

**Table 2:** Standard conditions for bioelectrical impedance analysis measurements

Patient status
Ensure a stable hydration state (avoid measurements after heavy fluid intake or during dehydration). No intense physical activity in the preceding 12 h. Fasting or no food intake for 4–6 h before the measurement. Avoid caffeine, alcohol, and tobacco several hours before testing. Empty the bladder before the measurement.
Time of measurement
Perform measurements at the same time of day, preferably in the morning, for repeated tests on the same individual.
Ambient temperature
Maintain a room temperature between 22–25 °C, as temperature affects fluid distribution and conductivity.
Patient position
Supine position (lying flat on the back) on a firm surface, with arms and legs slightly spread apart from the torso. Allow a rest period of at least 5–10 min before measurement to ensure fluid redistribution. Avoid position changes just before measurement.
Skin preparation
Avoid lotions or creams on contact areas. If needed, clean skin with isopropyl alcohol at electrode sites. Wipe the skin thoroughly with a cotton ball before placing the electrodes.
Electrode placement
Always measure on the same side of the body (typically the dominant or the right side) for consistency. Use always the same disposable adhesive electrodes with a skin-contact gel surface ≥400 mm2. Place one pair of signal and detection electrodes on the hand (base of the middle finger and wrist) and another on the foot (base of the middle toe and ankle). Be careful to keep ≥5 cm distance between the signal and detection electrodes.
Device used
Always use the same device model, properly calibrated. Record the measurement settings used (e.g. type of device, measurement frequency). Record the raw data (*Z* and PhA or *R* and Xc) to be able to use other predictive equations than the one provided by the device.

PhA, phase angle; *R*, resistance; Xc, reactance; *Z*, impedance.

### Interpretation and clinical integration

Once data are collected, a validated predictive equation must be applied that accounts for the patient's demographic (e.g. age, sex, and ethnicity) and clinical profile (e.g. renal failure, diabetes, and severe obesity) [4^▪▪^]. Importantly, BIA-derived values should always be interpreted within a comprehensive clinical context, including functional status, biochemical markers, and comorbidities, to support accurate and informed clinical decision-making [41].

## CONCLUSION

BIA is a practical and versatile tool for assessing body composition in both clinical and research settings. However, its reliability hinges on device specifications, electrode configuration, and strict adherence to standardized measurement protocols. In hospital settings, the preferred choice should be a portable MF-BIA device with a tetrapolar or octopolar configuration that allows for measurements in both standing and supine positions and provides access to raw impedance data, to enable the use of population-specific predictive equations. As BIA technology continues to evolve, greater transparency, methodological rigor, and regulatory compliance will be essential to ensure its optimal use in both clinical practice and scientific research.

## Acknowledgements

*None.*

### Financial support and sponsorship

*None.*

### Conflicts of interest

*There are no conflicts of interest.*

## REFERENCES AND RECOMMENDED READING

Papers of particular interest, published within the annual period of review, have been highlighted as:

▪ of special interest
▪▪ of outstanding interest
